# ‘The likes of me running and walking? No chance’: Exploring the perceptions of adult patients with bronchiectasis towards exercise

**DOI:** 10.1177/17423953221108223

**Published:** 2022-06-12

**Authors:** Holly Royle, Carol Kelly

**Affiliations:** 1 School of Health and Care Professions, 8629 University of Winchester, Winchester, UK; 2 Respiratory Research Centre, Health Research Institute, 6249 Edge Hill University, Ormskirk, UK

**Keywords:** exercise, bronchiectasis, respiratory, activity

## Abstract

**Objective:** To explore the views and experiences of adult patients with bronchiectasis towards exercise. **Methods:** Semi-structured interviews with ten patients with bronchiectasis were conducted to explore perceptions of exercise, potential barriers and facilitators of exercise. Inductive thematic analysis was used to identify key themes. **Findings:** Five main themes: 1. The language of exercise 2. Facilitators to exercise 3. Barriers to exercise 4. Exercise has a positive impact on health and life expectancy 5. Grief regarding loss of ability **Discussion:** Participants perceived exercise as positive, but there was variance regarding what this entailed. Findings suggest healthcare professionals should consider the language used when prescribing exercise and provide clarity for patients and reflect on their own role in advising on exercise. There were both common and differing barriers and facilitators to exercise between participants. Holistic needs and the identification of these potential barriers and facilitators to exercise could aid compliance. Further research is needed to explore generalisability and the effectiveness of behaviour change models to improve engagement with exercise.

## Introduction

Bronchiectasis is an irreversible chronic respiratory condition that has a significant impact on health, functional status and mortality.^1^ It is characterised by abnormally dilated airways, increased and subsequent pooling of secretions resulting in a vicious cycle of susceptibility to further tissue damage.^2,3^ It is thought that approximately five in every 1000 adults in the United Kingdom have bronchiectasis.^4^

Symptoms of bronchiectasis include significant sputum production, dyspnoea and fatigue. Complications include worsening lung function, reduced quality of life and life expectancy, and there is a large treatment burden for patients and their healthcare providers.^5^ Patients with bronchiectasis have been found to have reduced exercise tolerance and capacity which has a negative impact on quality of life.^6–8^ This patient group have also been found to be less active than those without bronchiectasis.^9^

Exercise has been found to reduce exacerbation frequency, increase exercise tolerance and improve quality of life in patients with bronchiectasis.^10^ Exercise has also been identified as a method of secretion clearance which is significant for patients with bronchiectasis, for which secretion retention is a hallmark.^11,12^ Reluctance to exercise in patients with chronic respiratory conditions leads to continued functional decline which further impacts symptoms and quality of life.^13,14^

Exercise is a key component in Pulmonary Rehabilitation (PR). British and European guidelines for bronchiectasis recognise the role of exercise and recommend that PR and advice to exercise regularly should be offered to individuals with bronchiectasis whose breathlessness affects their activities of daily living.^2,15^ However, in practise many patients with bronchiectasis have been found not to participate in PR.^15–18^

A 2015 Cochrane review reported low adherence to advised management strategies, including exercise, in patients with bronchiectasis, and a link between poor adherence and negative health outcomes.^16^ The European Multicentre Bronchiectasis Audit and Research Collaboration (EMBARC) found experts and patients concurred that establishing accessible methods and increasing adherence to PR are priorities for research.^19^

Lavery et al. (2007) and McCullough et al. (2014) identified reasons for poor adherence to treatment for patients with bronchiectasis were multifactorial and they emphasised the need to explore patient views in order to identify ways of improving adherence to treatment.^20,21^ In COPD several factors, including patient perceptions of symptoms and the impact of health care professionals, have been identified as affecting attitudes of patients to exercise,^22,23^ but no literature was found investigating patient views and factors affecting participation in exercise for patients with bronchiectasis.

In order to understand reasons why uptake of exercise is so poor and how we may introduce effective measures to increase uptake and adherence, we aimed to explore the views and experiences of adult patients with bronchiectasis towards exercise. Objectives of this study were: to explore the essence of exercise for a selected group of adult patients with bronchiectasis; to identify commonalities and divergences between patients; and to identify perceived real and/or potential barriers and facilitators to exercise. This study was undertaken with the intention to explore the essence of exercise in order to inform clinical practise in order to improve support and provision of care for patients with bronchiectasis.

## Methods

### Study design

A qualitative exploratory study was undertaken at a single site in the North West of England. Semi-structured interviews were carried out one-to-one by HR with ten adult participants with bronchiectasis.

### Patient and public involvement

The study rationale and design were discussed with members of local patient support groups, including those with a diagnosis of bronchiectasis, who felt that the need was clear, and the study design was appropriate.

### Ethics

Ethical permission was granted by the Health Research Authority's Research Ethics Committees (Study reference no. 235915, REC reference 18/LO/0600). A minimal risk of participant distress secondary to interview topics was mitigated by signposting participants to appropriate support groups if required. Participation was voluntary with informed consent and confidentiality and anonymity preserved. Data was collected and stored according to local policies and procedures. Interview transcripts were only available to the research team.

### Participants

Ten participants were recruited in a convenience sample through consultant-led respiratory clinics. Inclusion criteria were over 18 years of age, English speaking and had a diagnosis of bronchiectasis confirmed by high resolution computer tomography. Exclusion criteria included the presence of a communication impairment, and bronchiectasis secondary to cystic fibrosis.

### Data collection

An interview guide devised from related literature was used to facilitate face to face interviews which were conducted in a private room at the hospital site in spring 2018 (Appendix 1). This was designed based on the body of evidence discussed in the introduction.^20,22^ Interviews were planned to take up to one hour but were ended when their natural close had been reached.

Interviews were digitally audio-recorded and transcribed verbatim by HR, with data anonymised. After the tenth interview it was judged that further interviews were unlikely to add anything to the analysis and no further interviews were undertaken.^24^

### Data analysis and reflexivity

Thematic analysis was used to analyse data. This fitted in with the qualitative nature of the study and had the aim of articulating participants’ views accurately and in a useful format.^24^ This process was inductive, and themes were identified based on data analysed rather than any predetermined theory or structure.^25^

Informed by phenomenology, the intention was to gather detailed descriptive information from patients’ lived experiences without comparison to preconceived ideas.^25^

The researchers acknowledged the potential impact of their own opinions and views on many aspects of the study. HR acknowledged that one aspect in particular was her role as a physiotherapist It was vital to the rigour of the study that a process was followed to attempt to ‘bracket’ preconceptions of the researchers in order to preserve openness and transparency.^25^ Appendix 2 demonstrates the initial stage of ‘bracketing’ prior to starting the study, and a summary of the reflexive diary process. This was done via a three-stage process of reflexivity adapted from a structure proposed by Chan, Fung and Chien, 2013.^26^ Quality audit was undertaken by CK, to validate findings and add rigour to the data analysis, particularly given HR's status as a novice researcher.^25^

## Findings

### Demographics

The age of participants ranged between 18 and 84 years old, with a mean of 57.6 years, which helped enrich the findings. The gender split was equal. The participants involved were adults with bronchiectasis, with the length of time since diagnosis varying between 2 and 60 years (mean 20.1 years). The level of impact that the participants’ symptoms had on their daily life was varied e.g. difficulty with housework, limited ability to go shopping. The number of exacerbations leading to hospitalisation in the previous 12 months ranged between 0 and 2, with a mean of 0.6. Self-reported hours spent exercising per week ranged between 0 and 12 h, with a mean of 3.3 h.

The number of exacerbations and amount of exercise recorded for the participants was self-reported and therefore there may be limitations to accuracy. Participants who originally reported that they did 0 h per week exercising did during the course of the interview identify that they participated in some form of exercise. Seven out of ten participants recruited had co-existing respiratory conditions (five had a diagnosis of asthma and two had a diagnosis of COPD) meaning that their respiratory symptoms cannot be solely attributed to bronchiectasis. Bronchiectasis is not always found in isolation, and therefore data may be a more true representation of the population.^15,27^

Five main themes together with corresponding sub-themes were identified (Table 1). Longer extracts of the selected quotations can be found in Appendix 3. A thematic map can be found in Appendix 4.

**Table 1.** Themes and sub-themes.

**1. The language of exercise**
a) Exercise is more intense activity only b) Daily activities count as exercise
**2. Facilitators to exercise**
a) Enjoyment b) Pacing and adaptation c) Advice of healthcare professionals d) Self-motivation and life-long participation e) Social/family support
**3. Barriers to exercise**
a) Not achievable due to normal level of breathlessness b) Having an acute exacerbation c) Embarrassment regarding symptoms d) Fear of exacerbating breathlessness e) Time
**4. Exercise has a positive impact on health and life expectancy**
**5. Grief regarding loss of ability**

#### The language of exercise

There was discrepancy amongst participants regarding what constituted exercise which can be broadly split into two subthemes:

##### Exercise is intense activity

Participants perceived ‘exercise’ as relating to very intense workouts, such as going to the gym, suggesting that they could not engage because it is too high level:

‘*Exercise is lifting weights or something like that’* P1 page 2 line 27

‘*in the gym, that sort of stuff’* P2 page 1 line 27

This is of note because those who perceived that exercise was a very intensive activity, also demonstrated a reluctance to participate in physical activity because it was too demanding.

##### Daily activities count as exercise

In contrast, some participants identified daily activities as a form of exercise:

‘*All the exercise I have is looking after my husband at the moment…so that's a bit of exercise I suppose?’* P2 page 2 line 1–2

Others felt that their normal level of walking, particularly going to the shops was a form of exercise. Some participants who described exercise as being intense activity initially, later identified in their interviews that they did in fact participate in activities such as walking and chair-based exercises; they just hadn't thought of that as exercise. This finding highlights different perceptions of what exercise involves, and that the associations between the words ‘exercise’ and ‘activity’ were perceived very differently.

#### Facilitators to exercise

Factors were discussed which individuals felt helped their engagement in exercise and a number of shared facilitators to exercise were identified as outlined in the subthemes below:

##### Enjoyment

Some participants discussed the enjoyment and pleasure that exercise gives them. They identified enjoyment as being strongly positive, and many directly linked it as a fundamental influencer of their exercise participation.

‘*It's great. It's good- it's fun”* P6 page 1 line 38

##### Pacing and adaptation

Another factor identified by participants was the adaption of exercises and the use of pacing which was regarded as a necessity in order to keep as active as possible.

‘*It's just learning to adjust’* P9 page 2 line 16

This adjustment was discussed as being something instrumental in their ability to continue carrying out their day to day tasks and highlighted that even seemingly simple alterations allowed them to stay active.

##### Advice of healthcare professionals

Several participants interviewed reported having been given advice on exercise. All of these participants reported that this advice had been given to them by physiotherapists. Of those that had been given advice they reported that this was positive as it had increased their knowledge of what to do, and had encouraged them to participate:

‘*‘A physio…talked about exercise with me, in what I should try to do and what I shouldn't try to do’* P4 page 4 line 19–20

##### Self-motivation and life-long participation

Participants reported that the reasons they keep active are related to their self-motivation and/or personality. On asking what helped keep them active, responses included:

‘*Just for myself really’* P3 page 2 line 21

‘*I’m self-starting in a way’* P4 page 4, line 24

##### *‘*Social/family support

Individuals also discussed the ‘*social side’* (P6 page 1 line 36) of exercise as a motivational factor, as well encouragement from their family:

‘*My husband and the grandkids. They make me (exercise).’* P7 page 2 line 18

#### Barriers to exercise

Participants also identified factors that hindered their engagement with exercise, or stopped them engaging in exercise completely as outlined in the following subthemes:

##### Breathlessness

Breathlessness was identified as a limiting factor by almost all the participants. When asked whether they exercised, some responded strongly that they ‘*can't exercise’* because of their baseline level of breathlessness:

‘*The likes of me running and walking? No chance’* P1 Page 2 line 31

The words ‘can't’ and ‘no chance’ came up on more than one occasion, and the wording suggests a strong belief that they are unable to participate in exercise. Interestingly, all these individuals did later identify in the interview that they were able to complete some forms of exercise with the use of adaptation and pacing. Breathlessness came across as an emotive topic for most as they felt this restricted their ability to carry out their planned activities.

##### Having an acute exacerbation

In addition, several participants identified acute exacerbations as a limiting factor to partaking in exercise, and that they avoided exercising when unwell:

‘*If my chest isn't feeling good then I can't even consider exercising’* P4 Page 4 line 4

Again, the language used by participants was very poignant, describing not only distressing symptoms such as ‘wheezing’, but also that exacerbations were a ‘struggle’. Three participants stated that they ‘can't’ exercise when their breathing is ‘worse’, clearly conveying that they wouldn't even consider adapted exercising as an option when acutely unwell.

##### Embarrassment regarding symptoms

Exercise was identified as a factor that could cause symptoms such as coughing and clearing secretions. This raised strong feelings of embarrassment and frustration, and some suggested that they would avoid exercise, or at least some types of exercise, because of this:

‘*Coughing, which I feel like doing now! But I won't do it in front of people.’* P2 page 1 line 16

‘*I know that the breathing's going to become rapid and I’m going to have stuff coming off my chest because I’d rather not.’* P9 Page 2 line 20–22

Frustration when unable to participate in activities demonstrated the strength of feeling towards symptoms that were perceived as embarrassing, and there was concern about the perceptions of other people towards them.

##### Fear of exacerbating breathlessness

Another perceived barrier to exercise highlighted was fear of exacerbating their breathlessness:

‘*It can make you worse in the sense of your symptoms getting worse’* P8 page 2 line 1

Yet again, the language used to relate to this topic was very emotive and suggested that this was a key deterrent from exercising.

##### Time

Another identified barrier was difficulty finding time to exercise, particularly for those with work or family/caring commitments.

‘*I’m stuck in a way… with time, you know*’ P10 page 2 line 3

#### Exercise has a positive impact on health and life expectancy

Participants were aware that exercise would have a positive effect on their long-term pulmonary health, their general health, and their life expectancy.

The language used was quite robust, and a number of individuals associated exercising with staying alive, and conversely associated being sedentary with death. Overall there was a good understanding of the general benefits of exercising and its importance in the management of a chronic respiratory condition.

Most participants identified that they found that exercising directly had a positive effect on their breathing:

‘*I found when I was doing the ‘pulmonary thing’ (*PR*) I was breathing better’* P1 Page 3 line 10

Some also reported that they felt that exercise had wider positive effects, such as keeping them generally well and living longer:

‘*I find that aerobic exercise is good for me. It's the one thing that will keep me alive, as my chest degrades’* P4 page 3 line 4–5

‘*If you just lie down then you’re going to die aren't you?’* P5 page 2 line 1–2

#### Grief regarding loss of ability

One shared opinion was regret that having bronchiectasis had significantly reduced their ability to exercise and stay active:

*‘I loved walking and I can't walk anymore…I can't walk half of that now’* P5 Page 1 line 24–26

‘*It does really get to me after being so active’* P1 Page 2 line 13–14

There appeared to be a shared sense of loss, frustration, and disappointment. The expressions used were quite moving and reflected a shared sense of grief in loss of function and the impact on their self-efficacy.

The discussion regarding loss of function secondary to symptoms of bronchiectasis went beyond exercise participation, and many discussed the impact on their ability to be active in their home and family life.

## Discussion

This study is, to our knowledge, the first to explore patients with bronchiectasis’ perceptions of exercise. It identified that there were differing perceptions of what exercise actually entails between these patients with bronchiectasis, and that there were similarities and differences between identified barriers and facilitators to engaging with exercise.

Not all participants had been advised to exercise, and those that had were advised by physiotherapists only. Those that had been advised to exercise felt that this advice was helpful and increased the likelihood of their engagement in exercise. The World Health Organisation (WHO) published a European strategy for physical activity in 2015 highlighting the need for all healthcare professionals to increase their knowledge of benefits of physical activity in order to advise their patients.^28^ The NICE guidelines regarding physical activity advice highlight that the promotion of physical activity is the remit of any healthcare professional that have a role in offering lifestyle advice, rather than solely that of physiotherapists.^29^ This suggests that there may have been missed opportunities to advise these patients by healthcare professionals who had contact with them. Lack of advice from healthcare professionals could be due to a number of factors, including time and confidence in making recommendations regarding exercise to these patients.

In addition, there was a disparity amongst participants regarding what constituted exercise. This is important as it highlights that there is a need to reflect upon the language used by healthcare professionals when discussing exercise with patients. The word ‘exercise’ itself may be associated with preconceptions of high intensity that patients feel is not achievable. This therefore may be off putting for patients. Alternatively, patients may feel that ‘exercise’ encompasses their daily activities and therefore do not push themselves to improve their fitness by going above and beyond this. One of two participants in the study who had engaged in PR did not recognise the term when discussed. Oxley et al. (2019) also considered there to be a discrepancy between language used between healthcare professionals and patients with chronic lung disease, and identified that language used to discuss exercise and PR could have a great impact on patient management and self-management.^30^ Increased clarity from healthcare professionals when giving advice regarding exercise may increase patient engagement in effective and appropriate exercise.

Findings here also suggest that there are a number of similar facilitators and barriers to exercise between patients with bronchiectasis. These facilitators included enjoyment, self-motivation, adaptation, and importance of social/family support which could be used to inform strategies to increase adherence to exercise. Strategies involving some of these facilitators to increase uptake of exercise have been explored in other respiratory patient groups.^31,32^

Identified barriers to exercise, such as breathlessness, embarrassment regarding symptoms, and fear of exacerbating symptoms, suggests that healthcare professionals need to engage with their patients and discuss any potential concerns. Many participants identified feelings of grief regarding loss of functional ability and there was a link between impact of these feelings and self-efficacy. It has been suggested that self-efficacy directly has an impact on motivation and participation in exercise.^33,34^ Therefore, this could suggest a vicious cycle of inactivity leading to reduced self-efficacy, with this in turn leading to further inactivity. Lavery et al. (2007) also identified self-efficacy as an important part of compliance to managing long term conditions and suggest that this could be addressed by using health belief models and behaviour change models to increase adherence.^21^

Identification of, and emotive language used to describe both stigma regarding symptoms and grief regarding loss of previous fitness/function, suggests that discussions on engagement in exercise need to be open and holistic in approach. Of particular concern to some participants was stigma surrounding sputum expectoration, which is a hallmark of bronchiectasis. A systematic review of stigma-related experiences in other respiratory diseases found that perceived stigma and shame were associated with poor adherence to treatment, along with other adverse health behaviours.^35^ Though none of the included studies focussed on bronchiectasis, similar symptoms were reported. The authors highlighted that empathetic communication between patients and healthcare professionals can lead to higher patient satisfaction and improved health outcomes. Discussions and education by healthcare professionals therefore addressing areas of patient concern, may help patients’ perceptions of the risk/ benefit balance to exercise, and therefore may aid engagement.

### Strengths and limitations

In order to guide consideration of strengths and limitations of this study, the consolidated criteria for reporting qualitative research (COREQ) was used.^36^ One of the study's strengths is the attempt to enhance rigour and transparency throughout all phases and engagement with reflexivity. This will have reduced the impact of the researcher's own bias on data collection and analysis, therefore making the findings more valid.^37^

It is acknowledged that the use of public and ‘expert patient’ input could have been stronger. Patient involvement in research concerning the bronchiectasis patient group, e.g. in EMBARC, has had a great effect on the impact and quality of current research and guidelines and further involvement could have increased the impact of this study.^38^ In addition, the use of purposive sampling rather than convenience sampling, which was necessary due to limited time/resources, would have increased the credibility of the study and could have allowed a richer exploration of patient experience.^39^

Whilst this study cannot provide findings that can be generalised to the wider bronchiectasis population, it does highlight some aspects of current practise regarding advice on exercising that is worth reflecting on.

### Summary and implications for further research

Exercise was generally considered positive by participants, but perceptions of what this involved differed. This could be addressed by healthcare professionals considering their role and the language that they use in exercise prescription and then engaging in discussion with patients regarding their needs and/or concerns to facilitate engagement. Clarity also needs to be given to patients regarding what constitutes exercise. These findings suggest a number of shared and differing facilitators and barriers to exercise between patients with bronchiectasis. Further research needs to be carried out to identify whether these findings are generalisable to the wider bronchiectasis population and to investigate the effectiveness of behaviour change models to improve engagement with exercise in this patient group.

## Appendix 1: Interview guide

Introduction

- Hi, I am Holly Evans, the researcher for this study. Many thanks for agreeing to participate in this study, are you happy to continue? Can I confirm that you have read the PIS, had the opportunity to have any queries answered and signed the consent form? This interview is anticipated to take up to an hour, you are free to terminate the interview at any point if you wish. I will now ask you some questions.

1. Background

- When did you find out you had bronchiectasis?
- Have you ever been in hospital as a result of bronchiectasis? How many times?
- Tell me about any current treatment that you are receiving?

2. Quality of life

- Tell me about how bronchiectasis affects you?

Prompts:

- Does it affect your day to day life? How?
  - Does the impact it causes change?
  - Is there anything that it stops you doing?

3. Exercise

- What does the terms ‘exercise’ and ‘activity’ mean to you?

Prompts:

- What does it involve?
  - What do you think are different types of exercise or activity?

- Do you think people with bronchiectasis should exercise or stay active?

Prompts:

- Why/Why not?

- What do you think are possible effects of exercise and activity?

Prompts:

- Are there any positive effects?
  - Are there any negative effects?

- How physically active are you?

Prompts:

- How frequently do you exercise? For how long?
  - What type of exercise do you do?
  - Would you like to change this?

- Is there anything which limits how much exercise or activity you do?
- Is there anything that helps you stay active?
- Have you ever been given any advice on exercising or staying active?
- Have you ever heard the term ‘Pulmonary Rehabilitation’?

Prompts:

- Have you ever participated in this?
  - Tell me about it.

- Is there anything that you think would help you stay active or become more active?
- Is there anything else that we haven't covered that you would like to add?

End

Many thanks for your participation. Please retain the information sheet for your records, and feel free to contact myself via the contact details if you have any queries.

General interview prompts:

- Can you tell me a little more about that?
- In what way?
- Can you explain that?
- How is that?

## Appendix 2: Reflexivity stage 1: strategy for mental preparedness

**Stage 3: Reflexive diary**

A reflexive diary was kept during the data collection and analysis process. This involved reflections concerning HR's thoughts and expectations prior to, and after, each interview. This provided an opportunity to acknowledge and try to set aside HR's preconceptions. During the data collection process, it allowed HR to reflect on the interview questions and content, and the use of prompts, in order to try and minimise potential leading questions. During data analysis the bracketing mind map above was also revisited to further consider HR's preconceptions and to analyse the data more objectively. Many of the reflections concerned HR's pre-existing experiences treating patients with bronchiectasis, assumptions that there would be shared experiences for patients with bronchiectasis, HR’ role in prescribing exercise as a physiotherapist and HR's own understanding of the term ‘exercise’. Despite the attempts to minimise the impact of HR's own bias at various points in the study, the possible influence of this cannot be dismissed.

## Appendix 3: Participants quote extracts

**Participant 1**
**‘**Now to me, that wasn'*t exercise because.. going up and down stairs? I’ve got three steps into my house. So it's something I was doing all the time. So it's not exercise.. I don't class that as exercise. Exercise is lifting weights or something like that.’* Page 2 line 25–27

*‘I’d like to get back on the bike for exercise, but I can't.. I know I can't do it. I don't know about weights, lifting weights again, like I used to. And the likes of me running and walking? No chance.’* Page 2, line 29–31

*‘I found when I was doing the ‘pulmonary thing’; I was breathing better. If I stopped doing it (which I did a couple of times for different reasons) or if I sit on my backside too much, I start to find it difficult with my breathing. So to me, if I’m not exercising, my breathing goes worse.’* Page 3, line 10–13

*‘Well I can't do an awful lot that I used to do, because I used to do a lot of cycle rides, done the Mersey marathon you know. Cycle ride from Liverpool to Scarborough…the New Brighton one, the Birkenhead one, Liverpool to Chester- Liverpool to Manchester I should say. Liverpool to Southport. I was pretty fit in that way. Or even playing with the grandkids you know. Which is annoying. Or the likes of the grandson… Can't do it. But I’ll stand there and tell him what to do while he's doing it. But it does really get to me after being so active.’* Page 1, line 5–14

**Participant 2**
**‘**Well, that would be like, in the gym, that sort of stuff. Well, I’m 84 I don'*t think I’ll be doing that sort of stuff. I won't be doing a bit of exercise in the gym. No. All the exercise I have is looking after my husband at the moment, because he's bad.. he needs seeing to all the time. So that's a bit of exercise I suppose?’* Page 1 line 27

**Participant 3**
**‘**Just for myself really.’ Page 2 line 21

**Participant 4**
**‘**No because I’m self-starting in a way. I’ve always held the belief that if I can keep a little bit active it can help my chest I’ve always felt that. I’m sure there are people out there that feel that I can'*t do anything because my chest is so bad, that could benefit from what you’re doing by looking at this subject, and saying to them that they can improve their pulmonary health by exercising even with conditions like asthma and bronchiectasis.’*Page 4 line 24–28

*‘Yeh, a physio. They talked about exercise with me, in what I should try to do and what I should try to do. And when I should try to do it and when I shouldn't try to do it.’* Page 4 line 9–20

*‘If my chest isn't feeling good then I can't even consider exercising, because I’m working hard just to keep myself breathing and just day to day things.’* Page 4 line 4–5

*‘I find that aerobic exercise is good for me. It's the one thing that will keep me alive, as my chest degrades. It's something that I can't get rid of asthma and bronchiectasis- I can't get rid of them. There will be a degradation in my condition so I do try.’* Page 3 line 4–7

**Participant 5**
**‘**Oh it definitely works. It'*s definitely a must, exercise, when you’ve got this problem. If you just lie down then you’re going to die aren't you? So you’ve got to exercise.’* Page 2 line 1–2

*‘Since I’ve been diagnosed it has, I loved walking and I can't walk anymore. When I say I love walking, I was limited with my walking anyway- I have back trouble and all that you know. I couldn't walk far but I can't walk half of that now.’* Page 1 line 24–26

**Participant 6**
**‘**It'*s all positive really isn't it? You’ve got to keep active; you’ve got to keep moving. As we get older you just think ‘I can't be bothered’, but you’ve got to. It's the social side as well…it's great. It's good- it's fun.’* Page 1 line 35–38

**Participant 7**
**‘**My husband and the grandkids. They make me.’ Page 2, line 18

**Participant 8**
**‘**It can make you worse in the sense of your symptoms getting worse. On the other hand it helps clear your chest and makes you better. So there'*s two sides really.’* Page 2, line 1–2

**Participant 9**
**‘**It'*s just learning to adjust It's a bit of a nightmare when people ask you to do things and then you know you’ve got to sit out because you know you’re not going to be well enough to do it.’* Page 2 line 16–18

*‘When I’m sick I tend not to do as much because I know that the breathings going to become rapid and I’m going to have stuff coming off my chest because I’d rather not*’ Page 2 line 20–22

**Participant 10**
**‘**If I had the time, and I didn'*t have to look after my sister and other people you know. If I was on my own… I’ve always said I’d like to go back and swim… I keep saying I’ll do that, and go and do something like that, but I’m stuck in a way. With time, you know.’* Page 2, line 28–32

*‘Only for the time factor really. I’m looking after other people, so I don't really have the time. It stops me doing anything else’* Page 2, line 33–34
